# Proteomics-Based Approach to Identify Novel Blood Biomarker Candidates for Differentiating Intracerebral Hemorrhage From Ischemic Stroke—A Pilot Study

**DOI:** 10.3389/fneur.2021.713124

**Published:** 2021-12-17

**Authors:** David Malicek, Ilka Wittig, Sebastian Luger, Christian Foerch

**Affiliations:** ^1^Department of Neurology, Goethe University/University Hospital Frankfurt, Frankfurt am Main, Germany; ^2^Functional Proteomics, Institute of Cardiovascular Physiology, Faculty of Medicine, Goethe University, Frankfurt am Main, Germany

**Keywords:** mass spectrometry, blood, biomarker, differentiation, ischemic stroke, intracerebral hemorrhage

## Abstract

**Background:** A reliable distinction between ischemic stroke (IS) and intracerebral hemorrhage (ICH) is required for diagnosis-specific treatment and effective secondary prevention in patients with stroke. However, in resource-limited settings brain imaging, which is the current diagnostic gold standard for this purpose, is not always available in time. Hence, an easily accessible and broadly applicable blood biomarker-based diagnostic test differing stroke subtypes would be desirable. Using an explorative proteomics approach, this pilot study aimed to identify novel blood biomarker candidates for distinguishing IS from ICH.

**Material and Methods:** Plasma samples from patients with IS and ICH were drawn during hospitalization and were analyzed by using liquid chromatography/mass spectrometry. Proteins were identified using the human reference proteome database UniProtKB, and label-free quantification (LFQ) data were further analyzed using bioinformatic tools.

**Results:** Plasma specimens of three patients with IS and four patients with ICH with a median National Institute of Health Stroke Scale (NIHSS) of 12 [interquartile range (IQR) 10.5–18.5] as well as serum samples from two healthy volunteers were analyzed. Among 495 identified protein groups, a total of 368 protein groups exhibited enough data points to be entered into quantitative analysis. Of the remaining 22 top-listed proteins, a significant difference between IS and ICH was found for Carboxypeptidase N subunit 2 (CPN2), Coagulation factor XII (FXII), Plasminogen, Mannan-binding lectin serine protease 1, Serum amyloid P-component, Paraoxonase 1, Carbonic anhydrase 1, Fibulin-1, and Granulins.

**Discussion:** In this exploratory proteomics-based pilot study, nine candidate biomarkers for differentiation of IS and ICH were identified. The proteins belong to the immune system, the coagulation cascade, and the apoptosis system, respectively. Further investigations in larger cohorts of patients with stroke using additional biochemical analysis methods, such as ELISA or Western Blotting are now necessary to validate these markers, and to characterize diagnostic accuracy with regard to the development of a point-of-care-system for use in resource-limited areas.

## Introduction

In recent years, treatment options for patients with ischemic stroke (IS) have largely expanded. Subsequent to the broad implementation of intravenous thrombolysis, mechanical thrombectomy has now become the standard of care for patients with intracranial large vessel occlusion (1–5). Moreover, multimodal CT- and MR-imaging techniques allow the application of recanalizing treatment strategies even in extended time windows (6–9). In metropolitan areas, mobile stroke units have been released to apply thrombolysis already in the preclinical setting with the shortest possible delays after symptom onset (10, 11).

In contrast to these “high-tech” advances to stand by in many high-income countries, low- to middle-income countries still face immense shortcomings in medical resources. This weighs heavily as these countries have to carry the majority of the global burden of stroke (12). Regarding brain imaging, some countries have only one CT unit available per 1 million inhabitants (13). Hence, the stratification into IS and intracerebral hemorrhage (ICH) is not possible at all or only after long transports and transfer delays (14). This prevents acute target-orientated stroke treatment, but also from the timely initiation of diagnosis-specific secondary prevention (i.e., platelet inhibitors in patients with IS). However, the effect in reducing recurrent stroke for platelet inhibition is highest within the first weeks after the initial event (15).

Thus, in resource-limited areas, an inexpensive and easy-to-use stratification tool to substitute CT-imaging before the initiation of secondary prevention would be desirable. Here recent research on blood-based brain biomarkers has revealed interesting results. Glial fibrillary acidic protein (GFAP) has been characterized in several prospective studies as a biomarker of ICH. However, it reliably distinguishes ICH from IS only within 6 h of symptom onset. Its potential use has been demonstrated in an Indian trial, too (16–21). On the other side, comparable markers of IS have not been identified so far (22–24).

This pilot study aimed to identify candidate biomarkers suitable to differentiate IS and ICH within the first days after symptom onset. Hence, in an exploratory approach, the entire plasma/serum proteome was screened through mass spectrometry (MS) techniques. Ensuing, an extensive literature search was performed, to identify the relevant publications focusing on the diagnostic value of the candidate markers in acute stroke.

## Materials and Methods

### Study Design

For this explorative pilot study, we targeted to compare the two “prototypes” of stroke, i.e., patients with IS in the middle cerebral artery (MCA) territory and patients with ICH in the basal ganglia or the thalamus (“deep”) as well as in the parietal or temporal lobes (“lobar”). Both conditions typically present as a classical stroke syndrome, and differentiation between the entities solely based on clinical examination alone is usually not possible. To presume a considerable amount of brain tissue damage with release of brain proteins in the bloodstream, only patients with infarctions affecting at least one-third of MCA territory and only patients with hematoma volumes higher than 20 ml were included. For doing so, we screened plasma samples collected in the context of a prior prospective study on GFAP levels in neurological diseases performed in our center for these criteria (25). In total, plasma samples of three patients with IS and of four patients with ICH, who met the above criteria, were randomly chosen among the available samples. Ultimately, the cohort was enriched by serum samples from two healthy controls.

The Ethics Committee of the Goethe University Frankfurt am Main, Germany approved the protocols of the previous and the current study. The studies were conducted according to the principles of the Declaration of Helsinki. Written informed consent was obtained from each patient, or if applicable of the next-of-a-kin.

### Blood Sampling

According to the study protocol, 1 ml of ethylenediaminetetraacetic acid (EDTA)-plasma was collected during hospitalization at variable time points after stroke symptom onset and transferred into an Eppendorf tube (25). Within 60 min after blood draw, the samples were centrifugated at 10,000 g for 4 min, and the supernatant was immediately frozen and stored at −25°C; for long-term storing, the samples were transferred to −80°C freezers. Processing and storage of the serum samples of the two healthy controls were done in the same way.

### Mass Spectrometry

The protein content was determined by using the method of Lowry (26). For this, 200 μg of plasma/serum proteins were diluted to a final volume of 20 μl with 6 M GdmCl, 50 mM tris(hydroxymethyl)aminomethane (TRIS)/HCl, pH 8.5, 10 mM tris-carboxyethylphosphine (TCEP), and incubated at 95°C for 5 min. Reduced thiols were alkylated with 40 mM chloroacetamide and the samples were diluted with 25 mM TRIS/HCl, pH 8.5, 10% acetonitrile to obtain a final GdmCl concentration of 0.6 M. The proteins were digested with 2 μg trypsin (sequencing grade, Promega, WI, USA) overnight at 37°C under gentle agitation. Digestion was stopped by adding trifluoroacetic acid to a final concentration of 0.5%. The tryptic peptides were cleaned through reversed phase chromatography with C18 material (3M Empore™ SPE Extraction Disks) (27), dried in microtiter plates, and resolved in 1% acetonitrile and 0.1% formic acid before peptide identification.

Liquid chromatography/mass spectrometry (LC/MS) was performed on Thermo Scientific™ Q Exactive Plus equipped with an ultra-high-performance liquid chromatography unit (Thermo Scientific Dionex Ultimate 3000, Thermo Fisher Scientific, MA, USA) and a Nanospray Flex Ion-Source (Thermo Fisher Scientific, MA, USA). The peptides were eluted from the trap column by a continuously increasing concentration of organic solvent (4–50% acetonitrile and 0.1% formic acid) over 90 min at a flow rate of 250 nl/min and then, separated on an analytical column (with 2.4 μm Reprosil C18 resin from Dr. Maisch GmbH in-house packed picotip emitter tip with diameter 100 μm, 15 cm from New Objectives). The peptides were then ionized (2.6 kV) in the ion source and sprayed into the mass spectrometer. MS data were recorded by data dependent acquisition. The full MS scan range was 300–2,000 m/z with a resolution of 70,000, and an automatic gain control (AGC) value of 3 × 10^6^ total ion counts with a maximal ion injection time of 160 ms. Only higher charged ions (2+) were selected for MS/MS scans with a resolution of 17,500, an isolation window of 2 m/z, and an AGC value set to 10^5^ ions with a maximal ion injection time of 150 ms. MS-Data were acquired in profile mode, MS/MS data in Centroid mode. Each patient was measured one time. The two control donors were measured in technical triplicates and quadruplicates, respectively. All samples were measured consecutively with the same instrumental setup (identical analytical column, buffers, and mass calibration).

### MS Data Analysis

Mass spectrometry data were analyzed by MaxQuant (Max-Planck-Institute of Biochemistry, Martinsried, Germany) (v1.5.3.30) using default settings (28). Proteins were identified using the human reference proteome database UniProtKB with 71,567 entries, released in July 2017. The enzyme specificity was set to Trypsin. Acetylation (+42.01) at N-terminus, deamidation of N and Q (+0.98), and oxidation of methionine (+15.99) were selected as variable modifications and carbamidomethylation (+57.02) as a fixed modification on cysteines. False discovery rate (FDR) was calculated using the reverse decoy database implemented in MaxQuant. FDR was 1% for the identification of protein and peptides. Label-free quantification (LFQ) data were further analyzed using the bioinformatics tool Perseus (Max-Planck-Institute of Biochemistry, Martinsried, Germany) (v1.5.6.0) (29).

Contaminants from the internal MaxQuant list, only identified by site and reverse hits were removed from the initial protein ID list. The patients were grouped into ICH (*n* = 4) and IS (*n* = 3), the control group contains all the replicates (*n* = 7) of the two donors. Identified proteins were filtered to at least three valid values in one group. Missing values were replaced by the lowest value of the data set. A two-tailed Student's *t*-test was used to examine the levels of significance.

In addition, the statistics, correlations, and heat maps were created with Perseus as well. Other diagrams were created by using GraphPad Prism 8 (v.8.0.2) and an online Webtool from Bioinformatics and Evolutionary Genomics (http://bioinformatics.psb.ugent.be/webtools/Venn/).

### Review of the Literature

After identifying the biomarker candidates, we performed a structured literature search to identify relevant publications focusing on the diagnostic value of these markers in acute stroke. For doing so, a PubMed search was performed with the following search terms: the protein's name, its aliases, and its abbreviations according to UniProtKB (release 2021_01) linked (“AND”) to “stroke, apoplexy, ischemic stroke, ischaemic stroke, intracerebral hemorrhage or intracerebral haemorrhage.” Only studies published before August 2021 were included. Identified reviews were screened for primary sources. The results were filtered by the first author (DM) of this manuscript after a review of the title and abstract of the manuscripts. Publications relevant to the context of the present investigation were finally selected for evaluation. Please see the flow diagram (**Figure 3**) illustrating the database search for the review of the literature. The exact search terms are listed in the Supplementary Materials (Supplementary Table 5).

## Results

Mass spectrometry analysis comprised plasma samples of three patients with MCA infarction, four patients with ICH, and serum samples of two healthy controls (as outlined above in more detail). The baseline characteristics of the study population are depicted in Table 1.

**Table 1:** The baseline characteristics of the study subjects.

**Diagnosis**	**Sex**	**Age (years)**	**NIHSS**	**Time (days) between symptom onset and blood withdrawal**	**Protein content of the sample (mg/ml)**
Control	m	24	–	–	76.9
Control	m	27	–	–	69.5
IS	m	75	11	12 days	67.2
IS	m	69	10	8 days	60.8
IS	m	42	23	15 days	62.6
ICH	f	59	4	9 days	50.6
ICH	f	79	19	3 days	66.2
ICH	m	89	12	2 days	74.0
ICH	m	75	18	3.5 h	63.8

*IS, ischemic stroke; ICH, intracerebral hemorrhage; NIHSS, National Institute of Health Stroke Scale*.

Among the 495 identified protein groups, a total of 368 protein groups exhibited enough data points to be entered into quantitative analysis. Noticeably more proteins are expected to be present in human blood samples, however, proteins with low expression are strongly underrepresented in such analyses. Albumin, immunoglobulins, and transport proteins make up a large part and are increasingly represented in peptide analysis. Therefore, the tryptic peptides were separated by cation exchange chromatography. The serum/plasma amount of thus identified proteins is comparable with those reported in the literature (30). To validate the quality of data, the total results of all proteins were compared with those of all samples (Supplementary Figure 1). The determined correlation coefficients (> 0.88) show that the mass spectrometric analysis provides very homogeneous data (Supplementary Figure 2).

**Figure 1:**
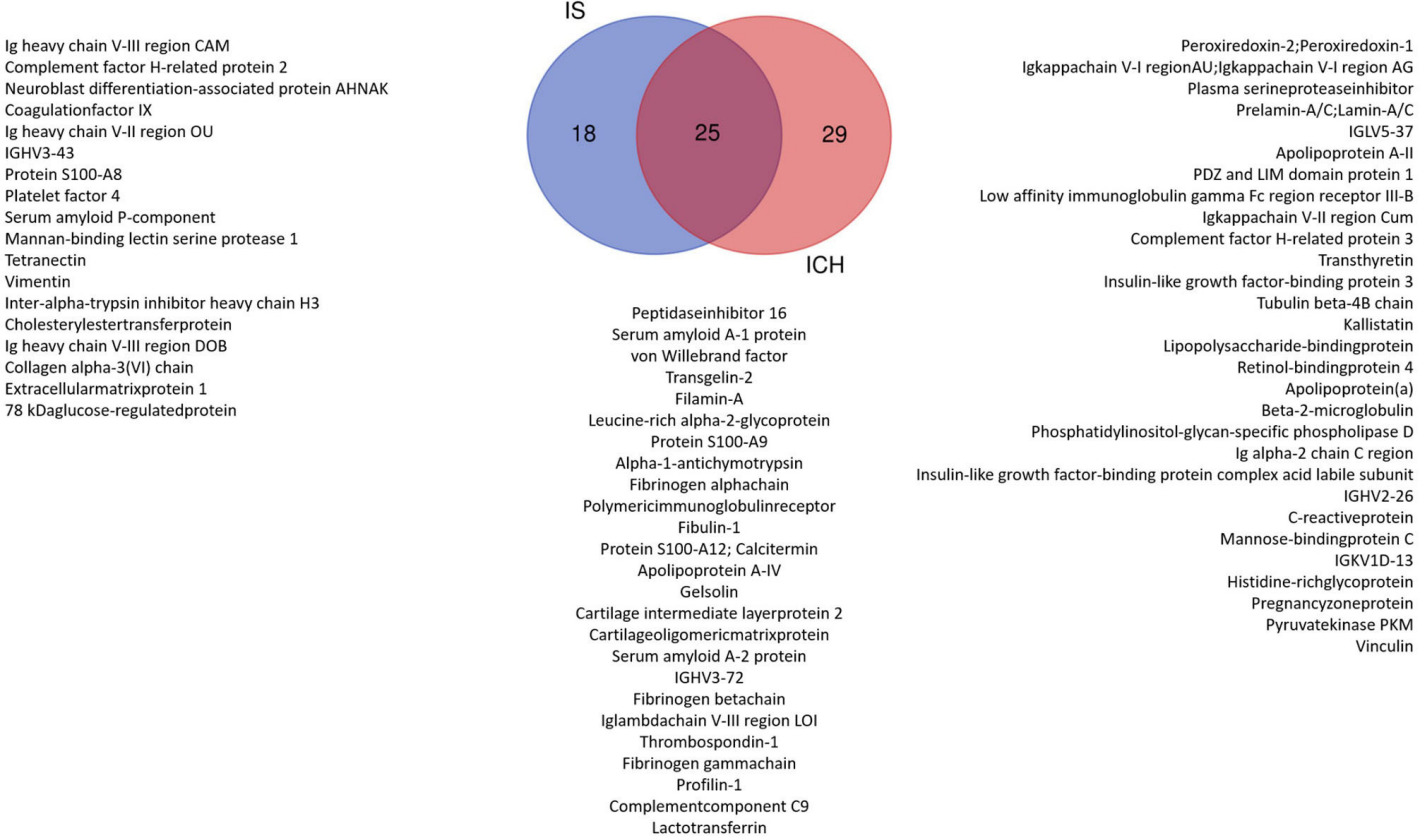
Venn diagram: significant proteins with at least 2-fold change in the patient group ICH vs. Control and IS vs. Control. We identified 25 proteins that were significantly changed in both the patient groups compared with Control. In total, 29 proteins were significantly different only in the ICH group and 18 only in the IS group.

Taking into account these 368 proteins, we then compared the amount of each protein in the patients group (P) as a whole (IS and ICH together) with the healthy (C) controls. Here, 91 proteins could be identified with significantly different amounts (P vs. C) (as shown in Supplementary Table 1; Supplementary Figure 3). In the next step, we compared the number of proteins in each disease entity, IS respectively ICH, with the healthy controls (C) (as shown in Supplementary Tables 2, 3). To create a top list of potential promising biomarkers, only proteins with significant differences with a fold change of at least ±2 between the groups (IS vs. C and ICH vs. C) were selected. Here, 25 proteins could be found both in the IS as well as in the ICH group with significant differences in abundance to the healthy controls, 18 individual proteins were differentially expressed only in the patients with IS, and 29 individual proteins only in the patients with ICH (as shown in Figure 1, Venn diagram).

Since the primary aim of the study was to identify differentially expressed proteins in patients with symptoms of stroke having either ICH or IS (and not necessarily in comparison with controls), we then screened for differences between ICH and IS, irrespective of differences to the control group and independent of the fold change, starting again from the original 368 proteins with enough data points (Supplementary Table 4). Here, 21 additional proteins could be identified (not part of the Venn diagram). After eliminating general structural and functional proteins, such as transport proteins, myosin chains, hemoglobin subunits, and components of immunoglobulins which are unsuitable as biomarkers due to their ubiquitous abundance, according to the literature (31, 32), 22 proteins remained and listed in Table 2.

**Table 2:** Protein “top list.”

**Protein**	**P vs. C**	**IS vs. C**	**ICH vs. C**	**IS vs. ICH**
Carboxypeptidase N subunit 2	ns	^**^	ns	^**^
Coagulationfactor XII	^**^	ns	^***^	^**^
Plasminogen	^**^	ns	^***^	^*^
Mannan-binding lectin serine protease 1	ns	^*^	ns	^*^
Serum amyloid P-component	^**^	^***^	^*^	^*^
Paraoxonase 1	ns	ns	ns	^*^
Carbonicanhydrase 1	ns	ns	ns	^*^
Fibulin-1	ns	^*^	ns	^*^
Granulins	ns	ns	ns	^*^
Inter-alpha-trypsin inhibitor heavy chain H3	^**^	^*^	^***^	ns
Coagulationfactor IX	^***^	^**^	^**^	ns
Protein S100-A8	^*^	^*^	ns	ns
Platelet factor 4	ns	^*^	ns	ns
C-reactive protein	^**^	ns	^**^	ns
Lipopolysaccharide-binding protein	^*^	ns	^**^	ns
Mannose-binding protein C	ns	ns	^*^	ns
Beta-2-microglobulin	^*^	ns	^**^	ns
PDZ and LIM domain protein 1	ns	ns	^*^	ns
Tubulin beta-4B chain	^*^	ns	^*^	ns
Pregnancy zone protein	^**^	ns	^**^	ns
Lamin-A/C	ns	ns	^*^	ns
Vinculin	^*^	ns	^*^	ns

*IS, ischemic stroke; ICH, intracerebral hemorrhage; C, healthy control; P, group of ICH + IS patients. ns (non-significant) p ≥ 0.05*,

* *p < 0.05*,

** *p < 0.01, and*

*** *p < 0.001; gray shaded are those proteins without significant difference between IS and ICH*.

### Candidate Proteins for Differentiating IS and ICH

**Figure 2:**
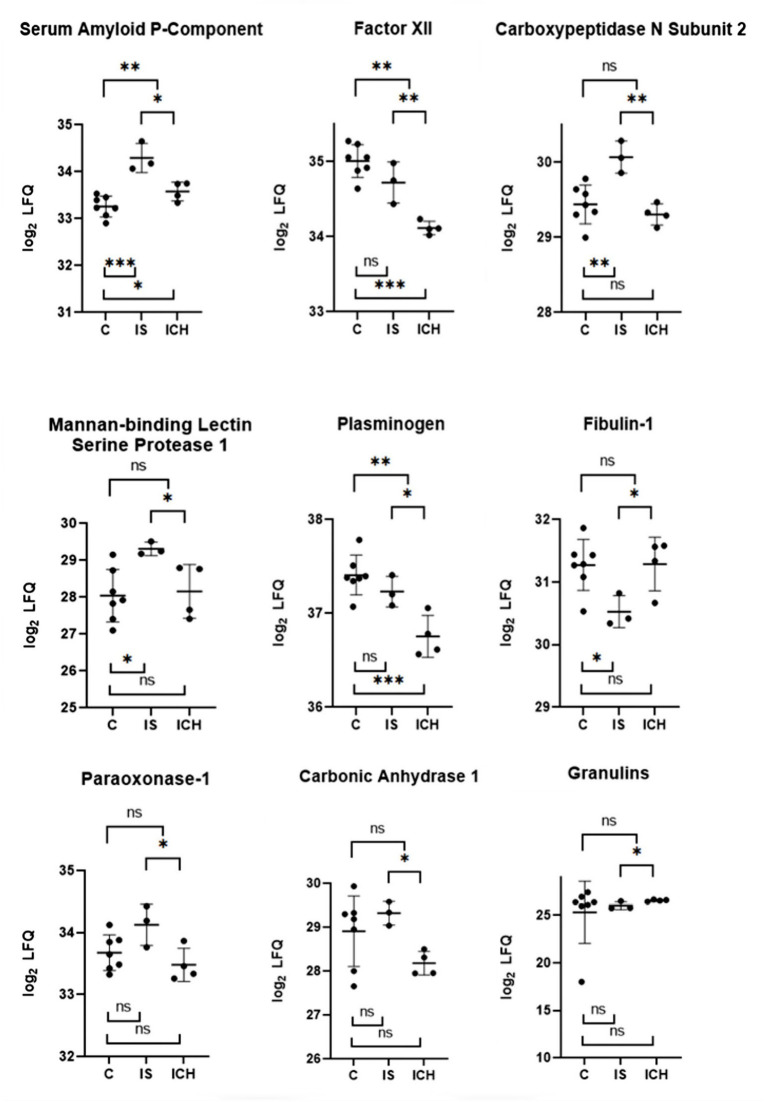
Selected proteins from the “top list” with the most significant differences within each group. The *y*-axis is transformed logarithmically. The scatter plots show the measured values with mean value and SD. Above the scatter plots, the differences between IS and ICH, respectively between the patients and control group are shown. Below the scatter plots, the differences between IS and control group are shown, as well as between ICH and control group. ns *p* ≥ 0.05, *p < 0.05, **p < 0.01, and ***p < 0.001.

Focusing on significant differences between the patients with ICH and IS, Figure 2 shows the nine most promising candidate proteins from the top list. Carboxypeptidase N subunit 2 (CPN2) and the coagulation factor XII (FXII) showed strongly increased (*p* < 0.01) protein amount in the patients with IS as compared with the patients with ICH. Both proteins play a role in the kinin-kallikrein-system and are involved in thrombus formation (33–36). Significantly increased (*p* < 0.05) protein amount in IS as compared to ICH patients were found for plasminogen (PLG), a central regulator in the fibrinolytic system, and Mannan-binding lectin serine protease 1 (MASP1), which was attributed a role in the blood clotting system by its thrombin-like activity (37, 38). Other proteins with a considerably increased amount of protein in IS in comparison to ICH patients, whose functionality is mainly not related to the blood coagulation system, were Amyloid P-component (APCS), Paraoxonase 1 (PON1), and Carbonic anhydrase 1 (CA1). Vice versa, only two proteins showed higher values in ICH patients as compared to IS patients [Fibulin-1 (FBLN1) and Granulines (GRN)].

### Review of the Literature and Integrative Evaluation

**Figure 3:**
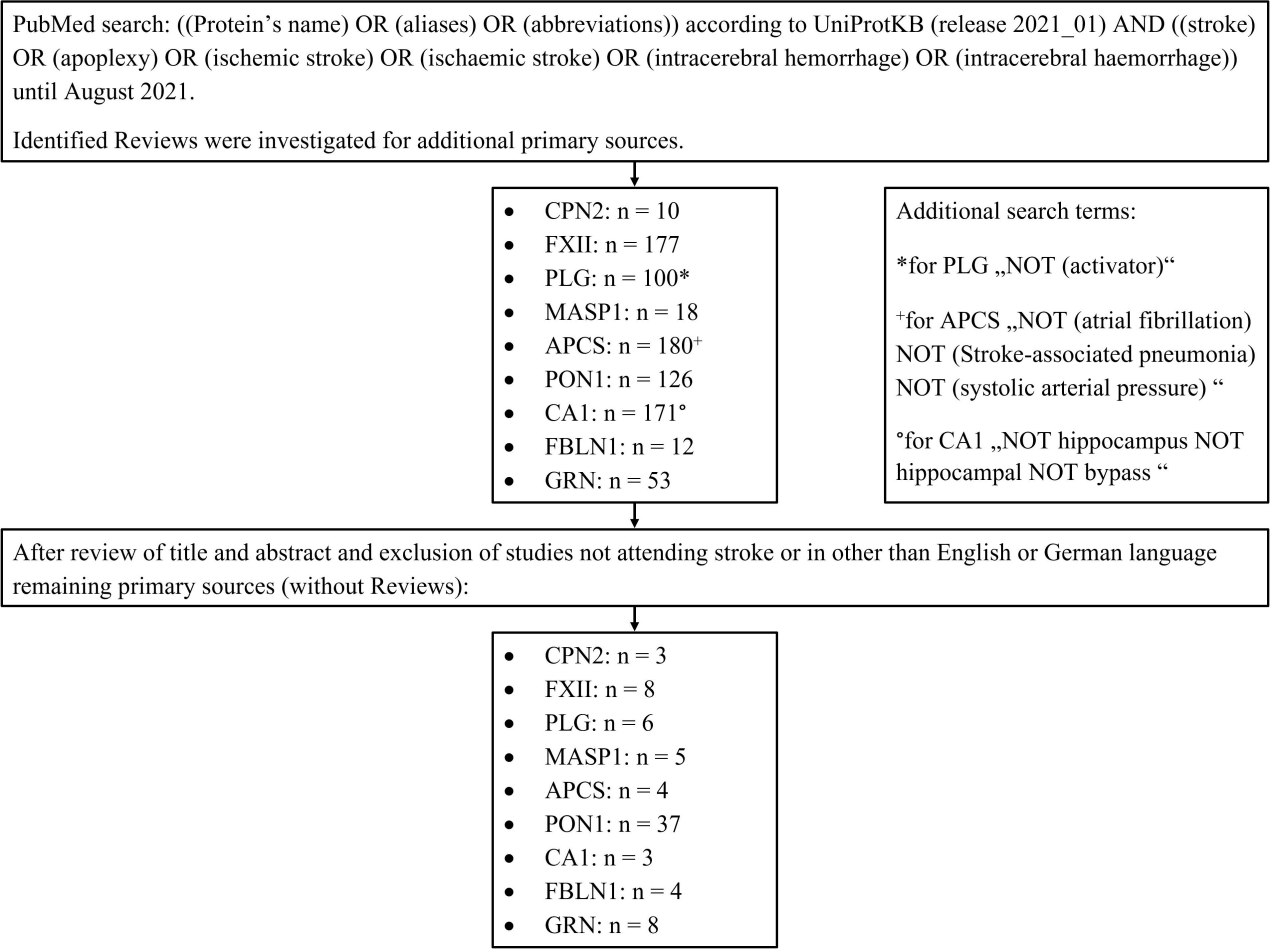
Flow diagram illustrating the database search for review of the literature.

After identifying the above outlined biomarker candidates, we reviewed the literature to figure out which proteins have already been investigated in human or animal studies relating to stroke and to integrate the current knowledge with our findings (as shown in Table 3; Figure 3). Overall, these studies analyzed patients with IS and ICH compared with controls, but not IS and ICH patients compared with each other. Moreover, the largest proportion of the available and identified studies analyzed serum and not plasma samples.

**Table 3:** Literature search.

**Protein**	**Species**	**Methodology**	**Collective**	**Summary of findings**	**References**
CPN2	Human	Mass spectrometry	Healthy	CPN is a component of fibrin-clots	(39, 40)
		Western blot			
	Human	Spectrophotometry	MI	Elevated CPN serum levels are detected in acute MI	(41)
FXII	Human	ELISA	IS and chronic cerebrovascular diseases	**Patients with chronic cerebrovascular disease show higher FXII levels in serum than patients with IS**	(42)
	Human	Medical hypothesis	IS	Via raised epinephrin levels due to chronic stress platelets activate pre-bound FXII which leads to hypercoagulability and together with essential hypertension favor atherosclerosis and ultimately IS	(43)
	Human	Case-control-study	IS	A certain gene polymorphism is a risk factor for IS	(44)
	Rat and Mouse	Neurological performance test	MCAO	Pharmacological inhibition of FXII reduces extent of infarction and improves neurological outcome after ischemia/reperfusion	(45, 46)
		Histopathology			
	Mouse	Histopathology	MCAO	FXII is essential for thrombus formation	(47–49)
		Immunofluorescence			
		Neurological performance test	Model of thrombo-embolism	Deficiency or inhibition of FXII protects from ischemic brain injury	
		MR			
PLG	Human	Mass spectrometry	IS	**IS patients show higher plasminogen blood levels than healthy subjects**	(50–52)
		Coagulation assay			
	Human	Chromozym assay	IS	**IS patients show lower plasminogen activity compared to healthy subjects**	(53)
	Human	Bioinformatical database research	IS	PLG was identified as critical protein for all subtypes of IS	(54)
	Mouse	Histopathology	Model of thrombo-embolism	Higher PLG levels attenuate brain infarction, endogenous fibrinolysis, microvascular thrombosis, inflammation, and BBB breakdown	(55)
		Immunofluorescence			
MASP-1	Human	Immunofluorimetry	IS and MI	**MASP1 shows higher levels in MI and lower levels in IS compared to controls**	(56)
	Human	Immunofluorescence	IS	**MASP-1 activity in IS patients is higher than in healthy subjects**	(57)
	Human	Immunofluorimetry	ICH and SAH	**MASP-1 levels decreased significantly in ICH and SAH patients during 24 h after symptom onset**	(58)
	Human	Immunofluorimetry	SAH	Cerebral blood concentration of MASP-1 is lower than in peripheral blood	(59)
	Mouse	Immunofluorescence	(FeCl_3_)-induced arterial thrombosis	MASP-1 has thrombin-like activity and is a significant regulator of thrombus formation *in vitro* and *in vivo*	(60)
		Immunostaining			
APCS	Human	Mass spectrometry	ICH	**Plasma APCS is higher in ICH than in healthy controls**	(61)
		Western Blot			
	Human	ELISA	Cardiovascular diseases	Increased APCS serum levels in the elderly are associated with angina pectoris and myocardial infarction but not with stroke	(62)
	Human	Mass spectrometry	Healthy	APCS is a component of fibrin-clots	(40)
	Human	Multiplex assay	IS	Increases in plasma levels of APCS are associated with worse clinical outcomes after IS	(63)
PON1	Human	ELISA	IS	**Serum PON1 activity is reduced in IS patients compared to controls**	(64–68)
		Spectrophotometry			
	Human	Multiplex assay	IS and ICH	**Serum PON1 activity is lower in IS patients than in ICH and controls**	(69)
	Human	Spectrophotometry	IS	PON-activity affects the outcome after IS	(70, 71)
	Human	Genetic engineering	IS	Particular gene polymorphisms (above all Q192R and L55M but also less common variants) and potentially the related enzyme activity raise the susceptibility for IS	(72–100)
CA1	Rat	Western Blot	ICH model	Erythrocyte lysis due to ICH may lead to CA release with tissue damaging and edema formation; Inhibition of CA reduces brain damage after ICH	(101)
	Rat	Mass spectrometry	ICH model	CA1 is upregulated in an ICH model compared to sham	(102)
	Human	Mass spectrometry	TBI & SAH	CA1 is elevated in CSF of TBI and SAH compared to controls, but no difference could be identified between TBI and SAH	(103)
FBLN1	Human	Mass spectrometry	IS	Serum FBLN1 is higher in a monozygotic twin suffering from IS	(104)
	Human	ELISA	Healthy	FBLN1 binds to Fibrinogen and is incorporated in Fibrin clots	(105)
	Human	Histopathology	Coronary heart disease	FBLN1 was detected in coronary atherosclerotic lesions and patients with unstable angina pectoris and acute MI show lower FBLN1 serum levels compared to controls	(106)
		Immunofluorescence			
	Human	Mass spectrometry	Cervical artery dissection-IS vs. Non-cervical artery dissection-IS	FBLN1 is significantly upregulated in IS due to cervical artery dissection compared to non-cervical artery dissection	(107)
		ELISA			
PGRN/GRN	Human	ELISA	IS	**Serum Progranulin levels are increased in IS compared to healthy controls**	(108)
	Human	ELISA	IS	GRN concentration affects outcome after IS	(109)
		Neurological performance test			
	Rat	Histopathology	Transient acute focal cerebral ischemia	Increased levels of PGRN expression in microglia within the ischemic core, increased levels of PGRN expression in viable neurons, induction of PGRN expression in endothelial cells within the ischemic penumbra	(110)
		Immunofluorescence			
	Rat	Histopathology	Transient acute focal cerebral ischemia	PGRN overexpression and artificial administration reduce cerebral infarction volume, edema, suppress hemorrhagic transformation and improve functional outcome	(110–114)
		Immunofluorescence			
	Mouse	Western Blot	MCAO		
	Mouse	Flow cytometry	MCAO	PGRN deficiency in mice leads to early BBB disruption and increased areas of hemorrhage in the ischemic territory	(115)
		Western Blot			
		Histopathology			
		Immunofluorescence			

*Investigation of protein levels in patients with stroke is highlighted in bold letters*.

Serum levels of CPN2 were not investigated in patients with stroke, but increased concentrations were found in patients with acute myocardial infarction (41). For FXII, studies showed high concentrations in patients with chronic cerebrovascular diseases (42), and FXII inhibition, as well as FXII deficiency improved outcome in experimental IS (45–49). In our study, FXII-levels were decreased in plasma of the patients with ICH compared with both controls and IS. For PLG, increased as well as decreased plasma levels in IS compared with controls have been described in the literature (50–53). We did not find differences between IS patients and controls, but ICH patients had significantly lower PLG protein amounts. MASP1 concentration was increased in myocardial infarction, however, in IS was reported to be either increased or decreased, whereas in ICH and SAH, lower levels were found (56–58). In our study, we detected higher MASP1 protein amount in IS than in healthy controls as well as in ICH. Consistent with our findings, one study showed higher APCS values in ICH than in controls (61). However, we additionally found higher values in IS compared to ICH patients. Among the identified proteins, PON1 seems to be best validated in human stroke patients, especially for several gene polymorphisms leading to higher susceptibility for IS. For PON1, reduced serum levels are described in IS (64–67, 69, 116). We did not find significant differences between IS and controls but within the patient groups elevated PON1 concentrations were found in IS compared to ICH. CA1 was found to be released from erythrocytes due to ICH in animal experiments but studies to evaluate blood levels of CA1 in patients with stroke are not available (101, 102). FBLN1 showed reduced serum concentrations in myocardial infarction (106). Comparable data for IS—except for the certain subgroup of IS caused by cervical artery dissection (107)—could not be identified. GRN was reported to be increased in the serum of patients with IS (108). It is expressed in the microglia in ischemic tissue (110–113). Increased GRN levels have not been confirmed in IS patients in our study.

### Most Promising Biomarker Candidates

Based on our data, we suggest that CPN2, FXII, PLG, MASP1, APCS, PON1, and CA1 for IS and FBLN1 and GRN for ICH should be further scientifically pursued as potential stroke blood biomarkers.

## Discussion

This exploratory pilot study identified nine proteins by means of mass spectrometry which showed different protein abundance in patients with IS and ICH. These nine proteins alone or in combination may now be evaluated in future prospective studies regarding their diagnostic accuracy to discriminate between those two subtypes of stroke. Moreover, detailed analyses of release kinetics of these markers after IS and ICH onset are mandatory.

At a closer look at the (patho-)physiological function of the identified proteins, it becomes apparent that all proteins, except APCS1 and CA1, are involved in the immune and/or coagulation system. This is not surprising because of previous findings on the pathophysiology of IS described as a “thrombo-inflammatory” disease (36, 117). The individual components of both systems influence each other. Besides its involvement in the coagulation cascade FXIIa activates the kinin-kallikrein system, which mediates, i.e., the activation of PLG and carboxypeptidases (35). Moreover, CPN seems to be involved in the kinin-kallikrein system and is able to reduce the cell binding capacity for PLG (33, 34, 37, 118). However, CPN itself is activated by plasmin as a negative feedback mechanism, ultimately leading to increased antifibrinolytic activity (119, 120). CPN, as well as FBLN1, have also been detected in fibrin clots (39, 106). By interaction of FBLN1 exposed after vascular injury with plasma fibrinogen, a linkage to a platelet integrin is formed and results in the formation of a platelet plug (105). CPN in the fibrin clot seems to act as a fibrinolysis inhibitor and belongs to the same family of zinc metalloproteinases as thrombin-activated fibrinolysis inhibitors (TAFI) (121). In turn, MASP1, traditionally attributed to the complement system, should interact with TAFIs, is conversely activated itself by activated platelets and fibrin and leads to fibrin clot formation and activation of thrombin and platelets, which seems to be essential for obstructive thrombosis at least in a mouse model of arterial injury (38, 60, 122). The formation of reactive oxygen species (ROS) in the context of ischemia/reperfusion processes with the influence of antioxidant components is reflected in the altered activity of associated proteins, such as PON1 (123, 124). Furthermore, the release of pro-inflammatory factors during IS leads to the activation of neuroprotective factors, such as GRN especially in viable neurons and endothelial cells in the ischemic penumbra (110, 112).

APCS and CA1 are not directly involved in coagulation or inflammatory pathways. APCS binds to apoptotic cells, is involved in chromatin degradation, and acts toxic to cerebral neurons (125, 126). CA1 is one of the 14 isoforms of carbonic anhydrases and occurs mainly in the cytosol of erythrocytes. In the context of ICH, erythrocyte lysis occurs around the hematoma, causing the release of iron and CA1 and subsequently increased tissue damage through edema formation and neuronal cell death. In addition, extracellular CA1 should promote the destruction of the blood-brain barrier by activating the kinin-kallikrein system (101, 127).

From a clinical point of view, until now no single biomarker identified in the context of stroke is suitable to certainly distinguish IS from ICH (21–24, 128). The most promising results so far have been published for GFAP. However, the different release kinetics of GFAP in IS and ICH exist only within the first 6 h after symptom onset (18, 129, 130). Thus, this protein is likely not helpful to differentiate strokes at any time point after symptom onset in resource limited settings. Moreover, GFAP release is strongly linked to the extent of damage to astroglial tissue. Thus, smaller ICH or expanding ICH may not always present with increased blood concentrations. More likely for this purpose, a combination of several markers may be favorable (131, 132). However, ischemic stroke is a heterogeneous disease comprising patients with large territorial infarctions and small lacunar strokes, as well as a large diversity of underlying etiologies. This makes it difficult to identify a biomarker panel that copes with all the facets of ischemic stroke. Interestingly, most of the proteins identified in our pilot study play pivotal roles in the immune and coagulation system. Thus, it is likely that they are directly involved in the pathophysiology of stroke and are not just an epiphenomenon. They are interesting candidates that add to the existing portfolio of potential biomarkers in stroke.

A shortcoming of this explorative pilot study is the very limited sample size. Reconfirmation of the core findings in larger patient cohorts is mandatory. In addition, other detection methods, such as ELISA or Western Blotting need to be applied to verify the proteins identified by mass spectrometry.

Furthermore, plasma samples were used for the primary comparison between IS and ICH. The control group, however, consisted of serum samples, thereby increasing the heterogeneity of the study.

Another limitation with the risk of a possible selection bias is the imbalance of baseline variables (such as age and sex) between the diagnosis groups. However, we focused on comparing “prototype” strokes as described above, and other exploratory studies based on the mass spectrometry techniques were designed in a similar way, nevertheless allowing the successful identification of novel biomarker candidates (133–135).

In summary, in this exploratory proteomics-based study, nine candidate blood biomarkers for differentiation of IS and ICH were identified. The proteins belong to the immune system, the coagulation cascade, and the apoptosis system, respectively. Due to the exploratory nature of the study, further investigations in independent, well-matched, and large-scaled cohorts of stroke patients are now necessary to validate these markers, and to characterize diagnostic accuracy with regard to the development of a point-of-care-system differentiating IS and ICH in resource-limited areas.

## Data Availability Statement

The raw data supporting the conclusions of this article will be made available by the authors, without undue reservation.

## Ethics Statement

The studies involving human participants were reviewed and approved by Ethics Committee of the Goethe University Frankfurt am Main. The patients/participants provided their written informed consent to participate in this study.

## Author Contributions

DM and IW: acquisition, analysis and interpretation of data, and drafting the work or revising it critically for important intellectual content. SL and CF: substantial contributions to the conception or design of the work and drafting the work or revising it critically for important intellectual content. All authors contributed to the article and approved the submitted version.

## Funding

This study was supported by the Deutsche Forschungsgesellschaft (DFG) project SFB815/Z1 (IW).

## Conflict of Interest

The authors declare that the research was conducted in the absence of any commercial or financial relationships that could be construed as a potential conflict of interest.

## Publisher's Note

All claims expressed in this article are solely those of the authors and do not necessarily represent those of their affiliated organizations, or those of the publisher, the editors and the reviewers. Any product that may be evaluated in this article, or claim that may be made by its manufacturer, is not guaranteed or endorsed by the publisher.

## Abbreviations

AGC: automatic gain control
APCS: serum amyloid P-component
BBB: blood brain barrier
CA1: carbonic anhydrase 1
CPN2: carboxypeptidase N subunit 2
CT: computed tomography
EDTA: ethylenediaminetetraacetic acid
ELISA: enzyme-linked immunosorbent assay
f: female
FDR: false discovery rate
Fig.: figure
FXII: factor XII
FBLN1: fibulin-1
GdmCl: guanidinium hydrochloride
GFAP: glial fibrillary acidic protein
GRN: granulins
HCl: hydrochloric acid
ICH: intracerebral hemorrhage
IQR: interquartile range
IS: ischemic stroke
LC-MS: liquid chromatography mass spectrometry
LFQ: label free quantification
m: male
MASP1: Mannan-binding lectin serine protease 1
MCA: middle cerebral artery
MCAO: middle cerebral artery occlusion
MI: myocardial infarction
MR: magnetic resonance
MS: mass spectrometry
m/z: mass-to-charge ratio
NIHSS: National Institutes of Health Stroke Scale
PLG: plasminogen
PON1: paraoxonase 1
Ref.: references
SAH: subarachnoid hemorrhage
Supp.: supplementary
TAFI: thrombin-activated fibrinolysis inhibitor
TBI: traumatic brain injury
TCEP: tris-carboxyethylphosphine
TRIS: tris(hydroxymethyl)aminomethane.
