# Supplementary material for: Proteomics-Based Approach to Identify Novel Blood Biomarker Candidates for Differentiating Intracerebral Hemorrhage From Ischemic Stroke—A Pilot Study
Source: Front Neurol. 2021 Dec 17;12:713124. doi: 10.3389/fneur.2021.713124 (PMC8719589; doi:10.3389/fneur.2021.713124)
Supplement: Supplementary file 1 [file Data_Sheet_1.docx]

Supplementary Material

# Supplementary Tables

**Supplementary Table 1**: Proteins with significantly different abundance in the patients group (IS and ICH) and the control group.

| Uniprot ID | Protein | Gene | Peptides | Student's T-test  P vs. C | Fold Change  P vs. C |
| --- | --- | --- | --- | --- | --- |
| P0DJI9 | Serum amyloid A-2 protein | SAA2 | 12 | 0.000024 | 2836.68 |
| P02741 | C-reactive protein | CRP | 7 | 0.008745 | 383.38 |
| P80511 | Protein S100-A12; Calcitermin | S100A12 | 1 | 0.000543 | 205.18 |
| P02679 | Fibrinogen gamma chain | FGG | 13 | 0.002002 | 183.50 |
| P21333 | Filamin-A | FLNA | 7 | 0.001944 | 147.21 |
| Q02985 | Complement factor H-related protein 3 | CFHR3 | 9 | 0.002695 | 110.10 |
| P01814 | Ig heavy chain V-II region OU | Ig heavy chain V-II region OU | 3 | 0.017908 | 98.51 |
| A0A0B4J2D9 | IGKV1D-13 | IGKV1D-13 | 2 | 0.005300 | 69.74 |
| P20742 | Pregnancy zone protein | PZP | 28 | 0.009566 | 51.44 |
| P37802 | Transgelin-2 | TAGLN2 | 6 | 0.006080 | 47.15 |
| P02675 | Fibrinogen beta chain | FGB | 23 | 0.002908 | 45.62 |
| I3L3D5 | Profilin-1 | PFN1 | 2 | 0.002900 | 43.52 |
| P0DJI8 | Serum amyloid A-1 protein | SAA1 | 13 | 0.000317 | 40.98 |
| P39060 | Collagen alpha-1(XVIII) chain; Endostatin | COL18A1 | 3 | 0.029421 | 25.81 |
| P18206 | Vinculin | VCL | 2 | 0.021920 | 24.19 |
| P40197 | Platelet glycoprotein V | GP5 | 7 | 0.043100 | 8.90 |
| P02788 | Lactotransferrin | LTF | 14 | 0.004250 | 7.57 |
| P29401 | Transketolase | TKT | 1 | 0.022078 | 7.24 |
| P01742 | Ig heavy chain V-I region EU | IGHV1-69-2 | 3 | 0.044783 | 6.11 |
| P02671 | Fibrinogen alpha chain | FGA | 28 | 0.000009 | 5.38 |
| P18428 | Lipopolysaccharide-binding protein | LBP | 9 | 0.015455 | 4.65 |
| P04275 | von Willebrand factor | VWF | 49 | 0.000311 | 3.91 |
| A0A0J9YY9; P01768 | Ig heavy chain V-III region CAM | Ig heavy chain V-III region CAM | 7 | 0.014175 | 3.87 |
| P01833 | Polymeric immunoglobulin receptor | PIGR | 7 | 0.001510 | 3.73 |
| P68371 | Tubulin beta-4B chain | TUBB4B | 2 | 0.024156 | 3.48 |
| P06702 | Protein S100-A9 | S100A9 | 12 | 0.009616 | 3.44 |
| P02750 | Leucine-rich alpha-2-glycoprotein | LRG1 | 21 | 0.000301 | 3.13 |
| P07996 | Thrombospondin-1 | THBS1 | 22 | 0.001501 | 2.93 |
| P05109 | Protein S100-A8 | S100A8 | 4 | 0.028036 | 2.56 |
| P02748 | Complement component C9 | C9 | 28 | 0.000017 | 2.53 |
| P01011 | Alpha-1-antichymotrypsin | SERPINA3 | 39 | 0.000306 | 2.26 |
| P61769 | Beta-2-microglobulin | B2M | 3 | 0.018391 | 2.15 |
| Q06033 | Inter-alpha-trypsin inhibitor heavy chain H3 | ITIH3 | 36 | 0.001062 | 1.98 |
| Q08380 | Galectin-3-binding protein | LGALS3BP | 10 | 0.001090 | 1.92 |
| P01877 | Ig alpha-2 chain C region | IGHA2 | 23 | 0.033121 | 1.91 |
| P00740 | Coagulation factor IX | F9 | 9 | 0.000463 | 1.86 |
| A0A0G2JPR0 | C4A | C4A | 127 | 0.023345 | 1.76 |
| P02763 | Alpha-1-acid glycoprotein 1 | ORM1 | 20 | 0.009391 | 1.68 |
| P00738 | Haptoglobin | HP | 50 | 0.024135 | 1.63 |
| A0A0G2JL69 |  | C2 | 28 | 0.002872 | 1.59 |
| P0C0L5 | Complement C4-B; C4a anaphylatoxin | C4B | 129 | 0.004467 | 1.58 |
| Q92954 | Proteoglycan 4 | PRG4 | 13 | 0.044010 | 1.58 |
| P01031 | Complement C5 | C5 | 57 | 0.001045 | 1.56 |
| P01034 | Cystatin-C | CST3 | 7 | 0.003395 | 1.55 |
| P02743 | Serum amyloid P-component | APCS | 12 | 0.005848 | 1.55 |
| Q15485 | Ficolin-2 | FCN2 | 6 | 0.033109 | 1.43 |
| P10643 | Complement component C7 | C7 | 34 | 0.028087 | 1.39 |
| P13671 | Complement component C6 | C6 | 44 | 0.004189 | 1.39 |
| P00450 | Ceruloplasmin | CP | 88 | 0.013661 | 1.39 |
| P00736 | Complement C1r subcomponent | C1R | 33 | 0.022865 | 1.36 |
| B7ZKJ8 | ITIH4 | ITIH4 | 61 | 0.026909 | 1.35 |
| P01009 | Alpha-1-antitrypsin | SERPINA1 | 65 | 0.021255 | 1.33 |
| P02746 | Complement C1q subcomponent subunit B | C1QB | 10 | 0.045013 | 1.29 |
| P04217 | Alpha-1B-glycoprotein | A1BG | 26 | 0.041890 | 1.28 |
| P00747 | Plasminogen | PLG | 78 | 0.008583 | -1.37 |
| P08185 | Corticosteroid-binding globulin | SERPINA6 | 7 | 0.015578 | -1.38 |
| P01825 | Ig heavy chain V-II region NEWM | IGHV4-61 | 4 | 0.043170 | -1.42 |
| P01042 | Kininogen-1; Bradykinin | KNG1 | 40 | 0.003082 | -1.43 |
| P02647 | Apolipoprotein A-I | APOA1 | 66 | 0.016569 | -1.48 |
| P02768 | Serum albumin | ALB | 149 | 0.000029 | -1.49 |
| P02787 | Serotransferrin | TF | 108 | 0.000175 | -1.54 |
| P00748 | Coagulation factor XII | F12 | 19 | 0.001972 | -1.55 |
| P51884 | Lumican | LUM | 13 | 0.000171 | -1.56 |
| P02654 | Apolipoprotein C-I | APOC1 | 7 | 0.034831 | -1.57 |
| P08697 | Alpha-2-antiplasmin | SERPINF2 | 22 | 0.000007 | -1.60 |
| A0A0G2JRQ6 | A0A0G2JRQ6 | A0A0G2JRQ6 | 1 | 0.046496 | -1.65 |
| P29622 | Kallistatin | SERPINA4 | 14 | 0.022338 | -1.65 |
| P08670 | Vimentin | VIM | 15 | 0.025616 | -1.70 |
| P80108 | Phosphatidylinositol-glycan-specific phospholipase D | GPLD1 | 18 | 0.013947 | -1.72 |
| P01599 | Ig kappa chain V-I region Gal | Ig kappa chain V-I region Gal | 4 | 0.002854 | -1.75 |
| P05090 | Apolipoprotein D | APOD | 13 | 0.005518 | -1.81 |
| P02765 | Alpha-2-HS-glycoprotein | AHSG | 16 | 0.000787 | -1.86 |
| P02753 | Retinol-binding protein 4 | RBP4 | 14 | 0.006264 | -1.90 |
| P02652 | Apolipoprotein A-II | APOA2 | 16 | 0.000001 | -1.99 |
| P05452 | Tetranectin | CLEC3B | 16 | 0.000004 | -2.06 |
| P02766 | Transthyretin | TTR | 16 | 0.001400 | -2.07 |
| P04196 | Histidine-rich glycoprotein | HRG | 26 | 0.000027 | -2.07 |
| Q16610 | Extracellular matrix protein 1 | ECM1 | 21 | 0.001701 | -2.25 |
| P06396 | Gelsolin | GSN | 57 | 0.000040 | -2.28 |
| P80748 | Ig lambda chain V-III region LOI | IGLV3-9 | 2 | 0.016704 | -2.29 |
| P49747 | Cartilage oligomeric matrix protein | COMP | 7 | 0.000356 | -2.30 |
| B1AHL2 | Fibulin-1 | FBLN1 | 14 | 0.013412 | -2.35 |
| P06727 | Apolipoprotein A-IV | APOA4 | 66 | 0.000269 | -2.76 |
| A0A0C4DH31 | IGHV1-18 | IGHV1-18 | 4 | 0.048325 | -2.86 |
| Q6UXB8 | Peptidase inhibitor 16 | PI16 | 6 | 0.000290 | -3.15 |
| P08519 | Apolipoprotein(a) | LPA | 40 | 0.007817 | -3.54 |
| P11021 | 78 kDa glucose-regulated protein | HSPA5 | 5 | 0.021679 | -7.88 |
| Q14623 | Indian hedgehog protein | IHH | 1 | 0.046108 | -8.46 |
| P05154 | Plasma serine protease inhibitor | SERPINA5; SERPINA3 | 4 | 0.047241 | -9.77 |
| P32119 | Peroxiredoxin-2; Peroxiredoxin-1 | PRDX2; PRDX1 | 2 | 0.031485 | -22.24 |
| Q8IUL8 | Cartilage intermediate layer protein 2 | CILP2 | 1 | 0.000012 | -134.63 |


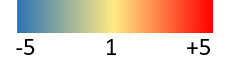


**Supplementary Table 2**: Proteins with significantly different abundance in the ICH group and the control group.

| Uniprot ID | Protein | Gene | Peptides | Student's T-test  ICH vs. C | Fold Change  ICH vs. C |
| --- | --- | --- | --- | --- | --- |
| P0DJI9 | Serum amyloid A-2 protein | SAA2 | 12 | 0.000026 | 6486.58 |
| P02741 | C-reactive protein | CRP | 7 | 0.001532 | 2383.01 |
| Q02985 | Complement factor H-related protein 3 | CFHR3 | 9 | 0.000000 | 576.65 |
| A0A0B4J2D9 | IGKV1D-13 | IGKV1D-13 | 2 | 0.000922 | 367.89 |
| P02679 | Fibrinogen gamma chain | FGG | 13 | 0.015561 | 191.14 |
| P20742 | Pregnancy zone protein | PZP | 28 | 0.009110 | 155.22 |
| P80511 | Protein S100-A12; Calcitermin | S100A12 | 1 | 0.010043 | 108.10 |
| P0DJI8 | Serum amyloid A-1 protein | SAA1 | 13 | 0.000234 | 70.29 |
| P21333 | Filamin-A | FLNA | 7 | 0.023406 | 61.67 |
| A0A075B6J1 | IGLV5-37 | IGLV5-37 | 1 | 0.033378 | 57.81 |
| P02675 | Fibrinogen beta chain | FGB | 23 | 0.019904 | 47.33 |
| P37802 | Transgelin-2 | TAGLN2 | 6 | 0.002792 | 37.23 |
| P18206 | Vinculin | VCL | 2 | 0.016891 | 36.64 |
| I3L3D5 | Profilin-1 | PFN1 | 2 | 0.023198 | 35.54 |
| O00151 | PDZ and LIM domain protein 1 | PDLIM1 | 5 | 0.019135 | 7.57 |
| P18428 | Lipopolysaccharide-binding protein | LBP | 9 | 0.002610 | 7.41 |
| P02788 | Lactotransferrin | LTF | 14 | 0.015588 | 7.33 |
| P68371 | Tubulin beta-4B chain | TUBB4B | 2 | 0.011916 | 4.94 |
| P02671 | Fibrinogen alpha chain | FGA | 28 | 0.000086 | 4.93 |
| P04275 | von Willebrand factor | VWF | 49 | 0.000950 | 4.30 |
| P02750 | Leucine-rich alpha-2-glycoprotein | LRG1 | 21 | 0.000057 | 3.95 |
| P01833 | Polymeric immunoglobulin receptor | PIGR | 7 | 0.008442 | 3.93 |
| P06702 | Protein S100-A9 | S100A9 | 12 | 0.028729 | 3.07 |
| Q3BDU5;  P02545; Q5TCI8 | Prelamin-A/C;Lamin-A/C | LMNA | 4 | 0.041714 | 2.82 |
| P02748 | Complement component C9 | C9 | 28 | 0.000135 | 2.64 |
| P07996 | Thrombospondin-1 | THBS1 | 22 | 0.020765 | 2.61 |
| P61769 | Beta-2-microglobulin | B2M | 3 | 0.006568 | 2.31 |
| P01011 | Alpha-1-antichymotrypsin | SERPINA3 | 39 | 0.000031 | 2.28 |
| P01594; P01593 | Ig kappa chain V-I region AU;Ig kappa chain V-I region AG | Ig kappa chain V-I region AU; Ig kappa chain V-I region AG | 3 | 0.033823 | 2.24 |
| P01877 | Ig alpha-2 chain C region | IGHA2 | 23 | 0.020207 | 2.18 |
| A0A0B4J1V2 | IGHV2-26 | IGHV2-26 | 3 | 0.026278 | 2.12 |
| P11226 | Mannose-binding protein C | MBL2 | 3 | 0.011946 | 2.05 |
| Q06033 | Inter-alpha-trypsin inhibitor heavy chain H3 | ITIH3 | 36 | 0.000893 | 1.96 |
| A0A0G2JPR0 | C4A | C4A | 127 | 0.012467 | 1.96 |
| Q08380 | Galectin-3-binding protein | LGALS3BP | 10 | 0.005286 | 1.94 |
| P02763 | Alpha-1-acid glycoprotein 1 | ORM1 | 20 | 0.002131 | 1.91 |
| P00738 | Haptoglobin | HP | 50 | 0.017795 | 1.76 |
| P00740 | Coagulation factor IX | F9 | 9 | 0.003660 | 1.70 |
| P01034 | Cystatin-C | CST3 | 7 | 0.016440 | 1.57 |
| P00450 | Ceruloplasmin | CP | 88 | 0.010251 | 1.49 |
| A0A0G2JL69 |  | C2 | 28 | 0.014511 | 1.46 |
| P01009 | Alpha-1-antitrypsin | SERPINA1 | 65 | 0.002589 | 1.41 |
| P01031 | Complement C5 | C5 | 57 | 0.011098 | 1.39 |
| P02746 | Complement C1q subcomponent subunit B | C1QB | 10 | 0.022458 | 1.37 |
| P0C0L5 | Complement C4-B; C4a anaphylatoxin | C4B | 129 | 0.037879 | 1.34 |
| P00736 | Complement C1r subcomponent | C1R | 33 | 0.025762 | 1.34 |
| K7ERG9; P00746 | Complement factor D | CFD | 5 | 0.032761 | 1.33 |
| P13671 | Complement component C6 | C6 | 44 | 0.047771 | 1.26 |
| P02743 | Serum amyloid P-component | APCS | 12 | 0.040485 | 1.25 |
| P02768 | Serum albumin | ALB | 149 | 0.000053 | -1.42 |
| P01042 | Kininogen-1; Bradykinin | KNG1 | 40 | 0.011722 | -1.49 |
| P08185 | Corticosteroid-binding globulin | SERPINA6 | 7 | 0.001616 | -1.51 |
| P00747 | Plasminogen | PLG | 78 | 0.000921 | -1.57 |
| A0A0G2JRQ6 | Ig-like domain-containing protein | A0A0G2JRQ6 | 1 | 0.002903 | -1.59 |
| P51884 | Lumican | LUM | 13 | 0.000266 | -1.61 |
| P02787 | Serotransferrin | TF | 108 | 0.000760 | -1.63 |
| P08697 | Alpha-2-antiplasmin | SERPINF2 | 22 | 0.000088 | -1.64 |
| P01599 | Ig kappa chain V-I region Gal | Ig kappa chain V-I region Gal | 4 | 0.028255 | -1.67 |
| P05090 | Apolipoprotein D | APOD | 13 | 0.002552 | -1.68 |
| P04433; A0A0A0MRZ8 | Ig kappa chain V-III region VG | IGKV3D-11 | 7 | 0.034087 | -1.72 |
| P00742 | Coagulation factor X;Factor X light chain;Factor X heavy chain;Activated factor Xa heavy chain | F10 | 7 | 0.016214 | -1.72 |
| O75882 | Attractin | ATRN | 14 | 0.006333 | -1.74 |
| E7END6; P04070; H7BYX9 | Vitamin K-dependent protein C;Vitamin K-dependent protein C light chain;Vitamin K-dependent protein C heavy chain;Activation peptide | PROC | 7 | 0.038452 | -1.74 |
| Q96KN2; J3KRP0 | Beta-Ala-His dipeptidase | CNDP1 | 9 | 0.019319 | -1.79 |
| O95445; Q5SRP5 | Apolipoprotein M | APOM | 7 | 0.034854 | -1.83 |
| P00748 | Coagulation factor XII | F12 | 19 | 0.000031 | -1.86 |
| A0A0B4J1X5; A0A075B7E8 | IGHV3-74; IGHV3OR16-13 | IGHV3-74; IGHV3OR16-13 | 8 | 0.039670 | -1.91 |
| P05452 | Tetranectin | CLEC3B | 16 | 0.000054 | -1.95 |
| Q16610 | Extracellular matrix protein 1 | ECM1 | 21 | 0.003216 | -1.95 |
| A0A075B6R9 | IGKV2D-24 | IGKV2D-24 | 5 | 0.042719 | -1.96 |
| P02765 | Alpha-2-HS-glycoprotein | AHSG | 16 | 0.002003 | -1.97 |
| P06310; A0A075B6S6 | Ig kappa chain V-II region RPMI 6410 | IGKV2D-30 | 4 | 0.023108 | -1.99 |
| P02652 | Apolipoprotein A-II | APOA2 | 16 | 0.000011 | -2.02 |
| P29622 | Kallistatin | SERPINA4 | 14 | 0.007631 | -2.05 |
| B1AHL2 | Fibulin-1 | FBLN1 | 14 | 0.019454 | -2.07 |
| P35858 | Insulin-like growth factor-binding protein complex acid labile subunit | IGFALS | 24 | 0.026631 | -2.09 |
| P49747 | Cartilage oligomeric matrix protein | COMP | 7 | 0.007432 | -2.12 |
| P04196 | Histidine-rich glycoprotein | HRG | 26 | 0.000175 | -2.14 |
| A0A087X0Q4; A0A087WW87; P01614 | Ig kappa chain V-II region Cum | IGKV2-40 | 6 | 0.044200 | -2.18 |
| P02753 | Retinol-binding protein 4 | RBP4 | 14 | 0.008441 | -2.19 |
| P80108 | Phosphatidylinositol-glycan-specific phospholipase D | GPLD1 | 18 | 0.001961 | -2.25 |
| A6XND1; A6XND0; P17936 | Insulin-like growth factor-binding protein 3 | IGFBP3 | 5 | 0.011358 | -2.27 |
| P02766 | Transthyretin | TTR | 16 | 0.001502 | -2.29 |
| P80748 | Ig lambda chain V-III region LOI | IGLV3-9 | 2 | 0.039165 | -2.48 |
| P06396 | Gelsolin | GSN | 57 | 0.000188 | -2.50 |
| A0A087WZR4; H0Y4U3; M9MML6; O75015 | Low affinity immunoglobulin gamma Fc region receptor III-B | FCGR3B | 6 | 0.014420 | -2.52 |
| A0A0B4J1Y9 | IGHV3-72 | IGHV3-72 | 8 | 0.030111 | -2.60 |
| Q6UXB8 | Peptidase inhibitor 16 | PI16 | 6 | 0.000013 | -2.77 |
| P06727 | Apolipoprotein A-IV | APOA4 | 66 | 0.002386 | -2.98 |
| H3BQ34; H3BTN5; P14618; H3BT25; H3BUW1; H3BTJ2; H3BR70; B4DNK4 | Pyruvate kinase;Pyruvate kinase PKM | PKM | 2 | 0.042767 | -4.30 |
| P08519 | Apolipoprotein(a) | LPA | 40 | 0.000044 | -6.50 |
| P05154 | Plasma serine protease inhibitor | SERPINA5 SERPINA3 | 4 | 0.010048 | -35.39 |
| P32119 | Peroxiredoxin-2; Peroxiredoxin-1 | PRDX2; PRDX1 | 2 | 0.019110 | -48.58 |
| Q8IUL8 | Cartilage intermediate layer protein 2 | CILP2 | 1 | 0.000000 | -264.59 |


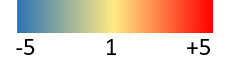


**Supplementary Table 3**: Proteins with significantly different abundance in the IS group and the control group.

| Uniprot ID | Protein | Gene | Peptides | Student's  T-test  IS vs. C | Fold Change  IS vs. C |
| --- | --- | --- | --- | --- | --- |
| P0DJI9 | Serum amyloid A-2 protein | SAA2 | 12 | 0.002842 | 941.61 |
| P80511 | Protein S100-A12; Calcitermin | S100A12 | 1 | 0.000158 | 482.16 |
| P21333 | Filamin-A | FLNA | 7 | 0.001498 | 469.60 |
| P01814 | Ig heavy chain V-II region OU | Ig heavy chain V-II region OU | 3 | 0.012284 | 400.66 |
| P02679 | Fibrinogen gamma chain | FGG | 13 | 0.032889 | 173.78 |
| P37802 | Transgelin-2 | TAGLN2 | 6 | 0.024087 | 64.60 |
| I3L3D5 | Profilin-1 | PFN1 | 2 | 0.030771 | 57.01 |
| P02675 | Fibrinogen beta chain | FGB | 23 | 0.035460 | 43.43 |
| P0DJI8 | Serum amyloid A-1 protein | SAA1 | 13 | 0.009705 | 19.96 |
| P01782 | Ig heavy chain V-III region DOB | Ig heavy chain V-III region DOB | 6 | 0.034288 | 9.05 |
| P02788 | Lactotransferrin | LTF | 14 | 0.014991 | 7.90 |
| P02671 | Fibrinogen alpha chain | FGA | 28 | 0.000447 | 6.05 |
| A0A0J9YY99; P01768 | Ig heavy chain V-III region CAM | Ig heavy chain V-III region CAM | 7 | 0.018711 | 4.17 |
| P06702 | Protein S100-A9 | S100A9 | 12 | 0.008580 | 4.01 |
| P01833 | Polymeric immunoglobulin receptor | PIGR | 7 | 0.002251 | 3.48 |
| P04275 | von Willebrand factor | VWF | 49 | 0.005772 | 3.45 |
| P07996 | Thrombospondin-1 | THBS1 | 22 | 0.002161 | 3.42 |
| P05109 | Protein S100-A8 | S100A8 | 4 | 0.030953 | 3.00 |
| A0A0B4J1Y9 | IGHV3-72 | IGHV3-72 | 8 | 0.021075 | 2.62 |
| P48740 | Mannan-binding lectin serine protease 1; Mannan-binding lectin serine protease 1 heavy chain; Mannan-binding lectin serine protease 1 light chain | MASP1 | 5 | 0.018647 | 2.41 |
| P02748 | Complement component C9 | C9 | 28 | 0.002483 | 2.40 |
| P02750 | Leucine-rich alpha-2-glycoprotein | LRG1 | 21 | 0.010155 | 2.30 |
| A0A0B4J1X8 | IGHV3-43 | IGHV3-43 | 4 | 0.030315 | 2.27 |
| P01011 | Alpha-1-antichymotrypsin | SERPINA3 | 39 | 0.009792 | 2.23 |
| P00740 | Coagulation factor IX | F9 | 9 | 0.003542 | 2.10 |
| P02743 | Serum amyloid P-component | APCS | 12 | 0.000292 | 2.05 |
| P02776 | Platelet factor 4; Platelet factor 4, short form | PF4 | 7 | 0.010635 | 2.03 |
| Q06033 | Inter-alpha-trypsin inhibitor heavy chain H3 | ITIH3 | 36 | 0.019036 | 2.01 |
| P55056 | Apolipoprotein C-IV | APOC4 | 5 | 0.040362 | 1.98 |
| P0C0L5 | Complement C4-B; C4a anaphylatoxin | C4B | 129 | 0.001282 | 1.97 |
| A0A0B4J1U7 | IGHV6-1 | IGHV6-1 | 4 | 0.015571 | 1.94 |
| Q08380 | Galectin-3-binding protein | LGALS3BP | 10 | 0.003009 | 1.91 |
| Q92954 | Proteoglycan 4 | PRG4 | 13 | 0.031080 | 1.86 |
| P09486; F5GY03 | SPARC | SPARC | 8 | 0.014229 | 1.85 |
| P01031 | Complement C5 | C5 | 57 | 0.002044 | 1.82 |
| A0A0G2JL69 | C3/C5 convertase | C2 | 28 | 0.011293 | 1.77 |
| P04430 | Ig kappa chain V-I region BAN | Ig kappa chain V-I region BAN | 1 | 0.009111 | 1.68 |
| Q13790 | Apolipoprotein F | APOF | 6 | 0.026147 | 1.66 |
| Q15485 | Ficolin-2 | FCN2 | 6 | 0.046261 | 1.61 |
| P04003 | C4b-binding protein alpha chain | C4BPA | 42 | 0.009297 | 1.59 |
| P13671 | Complement component C6 | C6 | 44 | 0.001652 | 1.58 |
| P15169 | Carboxypeptidase N catalytic chain | CPN1 | 11 | 0.036402 | 1.56 |
| P22792 | Carboxypeptidase N subunit 2 | CPN2 | 10 | 0.006194 | 1.55 |
| P01034 | Cystatin-C | CST3 | 7 | 0.042394 | 1.53 |
| A0A075B6P5; P01615 | Ig kappa chain V-II region FR | IGKV2D-28 | 4 | 0.002188 | 1.51 |
| G3XAM2; E7ETH0; P05156; A0A087X0I2 | Complement factor I;Complement factor I heavy chain; Complement factor I light chain | CFI | 26 | 0.027395 | 1.50 |
| P01024 | Complement C3; Complement C3 beta chain; Complement C3 alpha chain; C3a anaphylatoxin; Acylation stimulating protein; Complement C3b alpha chain | C3 | 188 | 0.028434 | 1.44 |
| P08571; D6RFL4 | Monocyte differentiation antigen CD14 | CD14 | 7 | 0.010816 | 1.42 |
| B4E1Z4; P00751 | Complement factor B; Complement factor B Ba fragment; Complement factor B Bb fragment | CFB | 87 | 0.049099 | 1.40 |
| P05155; H9KV48; E9PGN7 | Plasma protease C1 inhibitor | SERPING1 | 31 | 0.049389 | 1.25 |
| P80108 | Phosphatidylinositol-glycan-specific phospholipase D | GPLD1 | 18 | 0.032582 | -1.20 |
| A0A0B4J231; B9A064; A0A075B6K8; P0CG04 | Immunoglobulin lambda-like polypeptide 5; Ig lambda-1 chain C regions | IGLL5; IGLC1 | 16 | 0.043077 | -1.27 |
| P01834; A0A075B6H6 | Ig kappa chain C region | IGKC | 19 | 0.023662 | -1.35 |
| P01042 | Kininogen-1; Bradykinin | KNG1 | 40 | 0.016823 | -1.36 |
| A0A0A0MS08; P01857; A0A0A0MS07 | Ig gamma-1 chain C region | IGHG1 | 37 | 0.001184 | -1.40 |
| P02787 | Serotransferrin | TF | 108 | 0.000170 | -1.43 |
| P51884 | Lumican | LUM | 13 | 0.006074 | -1.48 |
| P14151 | L-selectin | SELL | 5 | 0.046102 | -1.50 |
| P01825 | Ig heavy chain V-II region NEWM | IGHV4-61 | 4 | 0.041792 | -1.53 |
| P08697 | Alpha-2-antiplasmin | SERPINF2 | 22 | 0.000119 | -1.54 |
| P02753 | Retinol-binding protein 4 | RBP4 | 14 | 0.020447 | -1.58 |
| P02768 | Serum albumin | ALB | 149 | 0.000885 | -1.59 |
| B0YIW2; P02656 | Apolipoprotein C-III | APOC3 | 10 | 0.000576 | -1.60 |
| A0A075B6K4 | IGLV3-10 | IGLV3-10 | 5 | 0.045999 | -1.66 |
| P23142 | Fibulin-1 | FBLN1 | 16 | 0.020301 | -1.68 |
| M0R0W6; P30530 | Receptor protein-tyrosine kinase; Tyrosine-protein kinase receptor UFO | AXL | 2 | 0.010008 | -1.70 |
| P02654 | Apolipoprotein C-I | APOC1 | 7 | 0.003488 | -1.72 |
| P02765 | Alpha-2-HS-glycoprotein | AHSG | 16 | 0.010719 | -1.73 |
| A0A075B7D8 | IGHV3OR15-7 | IGHV3OR15-7 | 5 | 0.021175 | -1.81 |
| P01876 | Ig alpha-1 chain C region | IGHA1 | 29 | 0.025901 | -1.81 |
| P02766 | Transthyretin | TTR | 16 | 0.014466 | -1.81 |
| P01599 | Ig kappa chain V-I region Gal | Ig kappa chain V-I region Gal | 4 | 0.004503 | -1.86 |
| P02647 | Apolipoprotein A-I | APOA1 | 66 | 0.002857 | -1.86 |
| P04432; P01597 | Ig kappa chain V-I region Daudi;  Ig kappa chain V-I region DEE | Ig kappa chain V-I region Daudi; Ig kappa chain V-I region DEE | 2 | 0.041404 | -1.90 |
| P02652 | Apolipoprotein A-II | APOA2 | 16 | 0.000350 | -1.95 |
| P05090 | Apolipoprotein D | APOD | 13 | 0.035308 | -1.99 |
| P04196 | Histidine-rich glycoprotein | HRG | 26 | 0.000805 | -1.99 |
| P06396 | Gelsolin | GSN | 57 | 0.001067 | -2.01 |
| P80748 | Ig lambda chain V-III region LOI | IGLV3-9 | 2 | 0.008381 | -2.07 |
| P11597 | Cholesteryl ester transfer protein | CETP | 1 | 0.004852 | -2.08 |
| P08670 | Vimentin | VIM | 15 | 0.038404 | -2.12 |
| P05452 | Tetranectin | CLEC3B | 16 | 0.000063 | -2.23 |
| P06727 | Apolipoprotein A-IV | APOA4 | 66 | 0.000113 | -2.50 |
| P49747 | Cartilage oligomeric matrix protein | COMP | 7 | 0.003095 | -2.56 |
| Q16610 | Extracellular matrix protein 1 | ECM1 | 21 | 0.003818 | -2.70 |
| Q09666 | Neuroblast differentiation-associated protein AHNAK | AHNAK | 2 | 0.020403 | -2.76 |
| B1AHL2 | Fibulin-1 | FBLN1 | 14 | 0.041899 | -2.79 |
| Q6UXB8 | Peptidase inhibitor 16 | PI16 | 6 | 0.004393 | -3.75 |
| P11021 | 78 kDa glucose-regulated protein | HSPA5 | 5 | 0.009703 | -17.43 |
| P36980; V9GYE7 | Complement factor H-related protein 2 | CFHR2 | 9 | 0.047393 | -24.13 |
| E7ENL6; P12111 | Collagen alpha-3(VI) chain | COL6A3 | 1 | 0.040705 | -25.97 |
| Q8IUL8 | Cartilage intermediate layer protein 2 | CILP2 | 1 | 0.003125 | -54.69 |


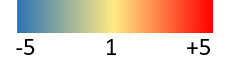


**Supplementary Table 4**: Proteins with significantly different abundance in the ICH group and the IS group.

| Uniprot ID | Protein | Gene | Peptides | Student's  T-test  ICH vs. IS | Fold Change  ICH vs. IS |
| --- | --- | --- | --- | --- | --- |
| P08590 | Myosin light chain 3 | MYL3 | 3 | 0.000003 | 66.63 |
| P10916 | Myosin regulatory light chain 2, ventricular/cardiac muscle isoform; Myosin regulatory light chain 10 | MYL2; MYL10 | 4 | 0.000002 | 41.74 |
| P35579 | Myosin-9 | MYH9 | 7 | 0.047758 | 11.38 |
| P01594; P01593 | Igkappachain V-I regionAU; Igkappachain V-I region AG | Igkappachain V-I regionAU; Igkappachain V-I region AG | 3 | 0.016600 | 2.96 |
| P23142 | Fibulin-1 | FBLN1 | 16 | 0.042941 | 1.70 |
| P01857 | Ig gamma-1 chain C region | IGHG1 | 37 | 0.010454 | 1.59 |
| P28799 | Granulins | GRN | 1 | 0.048806 | 1.47 |
| P00747 | Plasminogen | PLG | 78 | 0.026337 | -1.39 |
| P00748 | Coagulationfactor XII | F12 | 19 | 0.007977 | -1.52 |
| P27169 | Serum paraoxonase/arylesterase 1 | PON1 | 17 | 0.036158 | -1.56 |
| P02743 | Serum amyloid P-component | APCS | 12 | 0.013262 | -1.64 |
| P22792 | Carboxypeptidase N subunit 2 | CPN2 | 10 | 0.002240 | -1.70 |
| P04430 | Igkappachain V-I region BAN | Igkappachain V-I region BAN | 1 | 0.014411 | -1.78 |
| P69905 | Hemoglobinsubunitalpha | HBA1; HBA2 | 14 | 0.020431 | -1.79 |
| P68871 | Hemoglobin subunit beta; LVV-hemorphin-7; Spinorphin | HBB | 19 | 0.001696 | -1.88 |
| P04433; A0A0A0MRZ8 | Igkappachain V-III region VG | IGKV3D-11 | 7 | 0.021189 | -2.08 |
| P00915; E5RH81; E5RFE7; E5RHP7 | Carbonicanhydrase 1 | CA1 | 5 | 0.002775 | -2.20 |
| P48740 | Mannan-bindinglectinserineprotease 1 | MASP1 | 5 | 0.046516 | -2.23 |
| P02042 | Hemoglobinsubunitdelta | HBD | 12 | 0.008945 | -2.26 |
| A0A0B4J1Y9 | IGHV3-72 | IGHV3-72 | 8 | 0.015234 | -6.82 |
| P01782 | Ig heavy chain V-III region DOB | Ig heavy chain V-III region DOB | 6 | 0.034040 | -6.91 |

**Supplementary Table 5:** Pubmed search terms for “Review of the literature”

| CPN2 | ((Carboxypeptidase N) OR (Carboxypeptidase N subunit 2) OR (Carboxypeptidase N 83 kDa chain) OR (Carboxypeptidase N large subunit) OR (Carboxypeptidase N polypeptide 2) OR (Carboxypeptidase N regulatory subunit) OR (ACBP) OR (CPN2)) AND ((stroke) OR (apoplexy) OR (ischemic stroke) OR (ischaemic stroke) OR (intracerebral hemorrhage) OR (intracerebral haemorrhage)) |
| --- | --- |
| FXII | ((Coagulation factor XII) OR (F12) OR (Hageman factor) OR (HAF)) AND ((stroke) OR (apoplexy) OR (ischemic stroke) OR (ischaemic stroke) OR (intracerebral hemorrhage) OR (intracerebral haemorrhage)) |
| PLG | ((Plasminogen) OR (PLG)) AND ((stroke) OR (apoplexy) OR (ischemic stroke) OR (ischaemic stroke) OR (intracerebral hemorrhage) OR (intracerebral haemorrhage)) NOT (activator) |
| MASP1 | ((Mannan-binding lectin serine protease 1) OR (Complement factor MASP-3) OR (Complement-activating component of Ra-reactive factor) OR (Mannose-binding lectin-associated serine protease 1) OR (Mannose-binding protein-associated serine protease) OR (Ra-reactive factor serine protease p100) OR (RaRF) OR (CRARF) OR (CRARF1) OR (PRSS5) OR (MASP1)) AND ((stroke) OR (apoplexy) OR (ischemic stroke) OR (ischaemic stroke) OR (intracerebral hemorrhage) OR (intracerebral haemorrhage)) |
| APCS | ((Serum amyloid P-component) OR (SAP) OR (APCS) OR (9.5S alpha-1-glycoprotein) OR (PTX2)) AND ((stroke) OR (apoplexy) OR (ischemic stroke) OR (ischaemic stroke) OR (intracerebral hemorrhage) OR (intracerebral haemorrhage)) NOT (atrial fibrillation) NOT (Stroke-associated pneumonia) NOT (systolic arterial pressure) |
| PON1 | ((Serum paraoxonase/arylesterase 1) OR (Serum paraoxonase 1) OR (Serum arylesterase 1) OR (PON1) OR (Aromatic esterase 1) OR (Serum aryldialkylphosphatase 1)) AND ((stroke) OR (apoplexy) OR (ischemic stroke) OR (ischaemic stroke) OR (intracerebral hemorrhage) OR (intracerebral haemorrhage)) |
| CA1 | ((Carbonic anhydrase 1) OR (CA1) OR (Carbonate dehydratase) OR (Carbonic anhydrase B) OR (CAB) OR (CA-I)) AND ((stroke) OR (apoplexy) OR (ischemic stroke) OR (ischaemic stroke) OR (intracerebral hemorrhage) OR (intracerebral haemorrhage)) NOT (hippocampus) NOT (hippocampal) NOT (bypass) |
| FBLN1 | ((Fibulin-1) OR (Fibulin 1) OR (FIBL-1) OR (FBLN1)) AND ((stroke) OR (apoplexy) OR (ischemic stroke) OR (ischaemic stroke) OR (intracerebral hemorrhage) OR (intracerebral haemorrhage)) |
| PGRN/GRN | ((Progranulin) OR (Granulin) OR (Epithelin precursor) OR (Proepithelin) OR (Acrogranin) OR (PGRN) OR (GRN)) AND ((stroke) OR (apoplexy) OR (ischemic stroke) OR (ischaemic stroke) OR (intracerebral hemorrhage) OR (intracerebral haemorrhage)) |

# Supplementary Figures


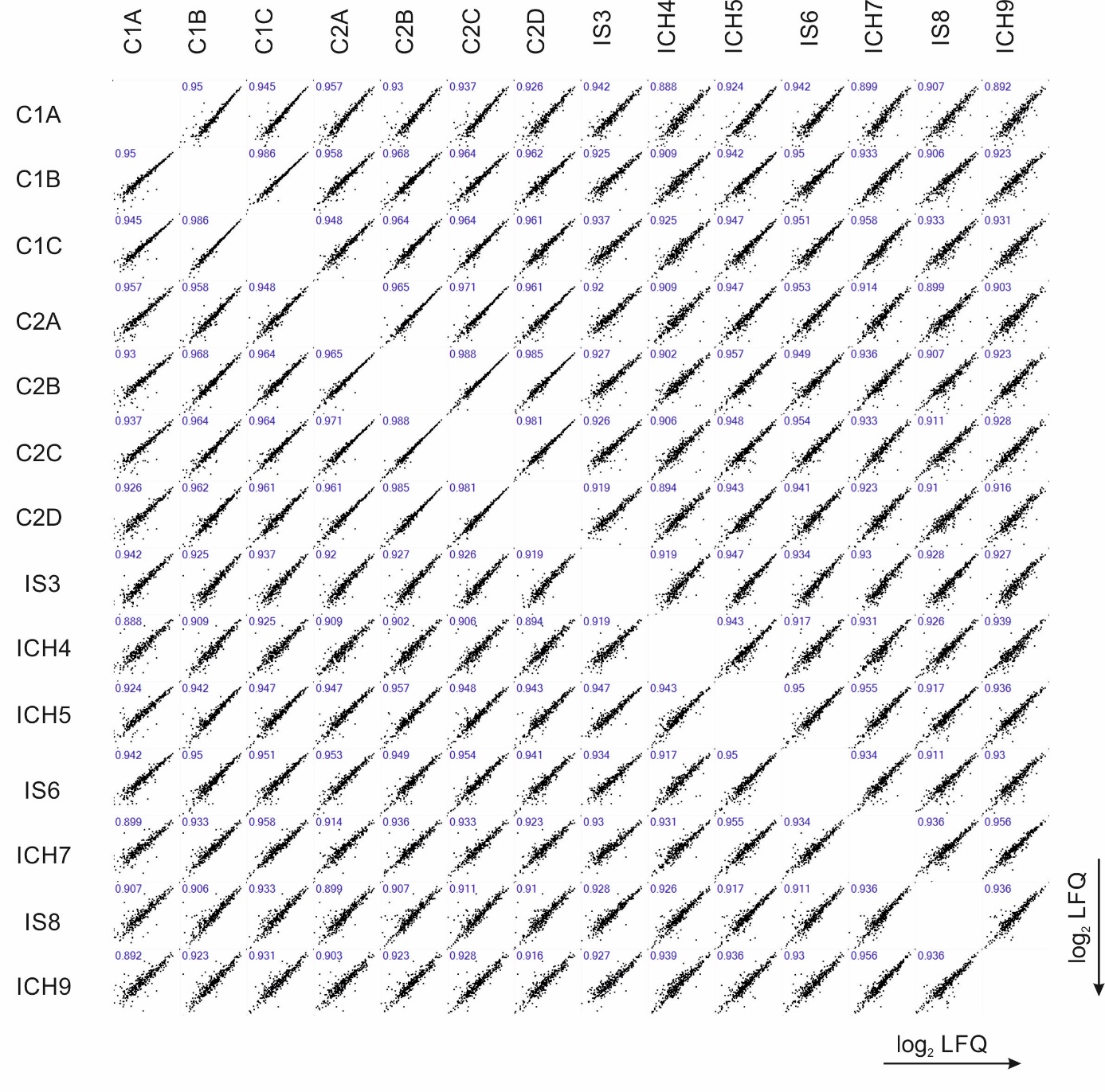


**Supplementary Figure 1.** **A matrix of scatter plots of log2 label-free protein values ​​from the mass spectrometric protein analysis.**

C1 ABC and C2 ABCD: Replicates of control 1 and 2 serum samples; IS 3,6,8: Ischemic stroke serum samples; ICH 4,5,7,9: Intracerebral hemorrhage serum samples. The number within each scatter plot represent the Pearson’s correlation value for each plot. Equal amounts of protein collect as a “diagonal” in the scatter plot and show the high correlation of the protein amount. It is assumed that human sera in their protein entirety do not differ much.


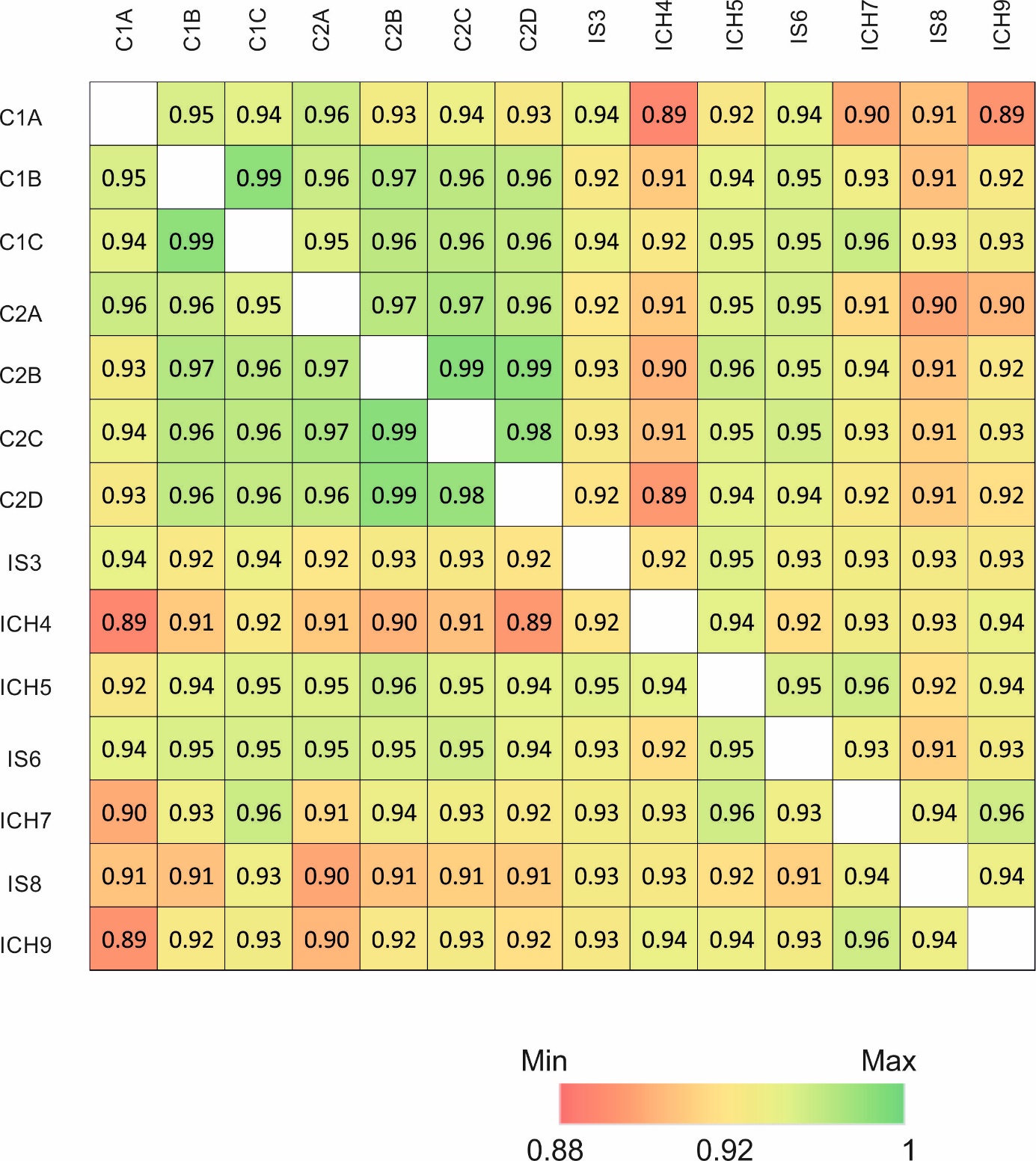


**Supplementary Figure 2.** **Heat map of** **Pearson’s correlation coefficients regarding the protein amount between the individual samples.**

With correlation coefficients > 0.88, the mass spectrometric analysis provided very homogeneous data with high quality results.


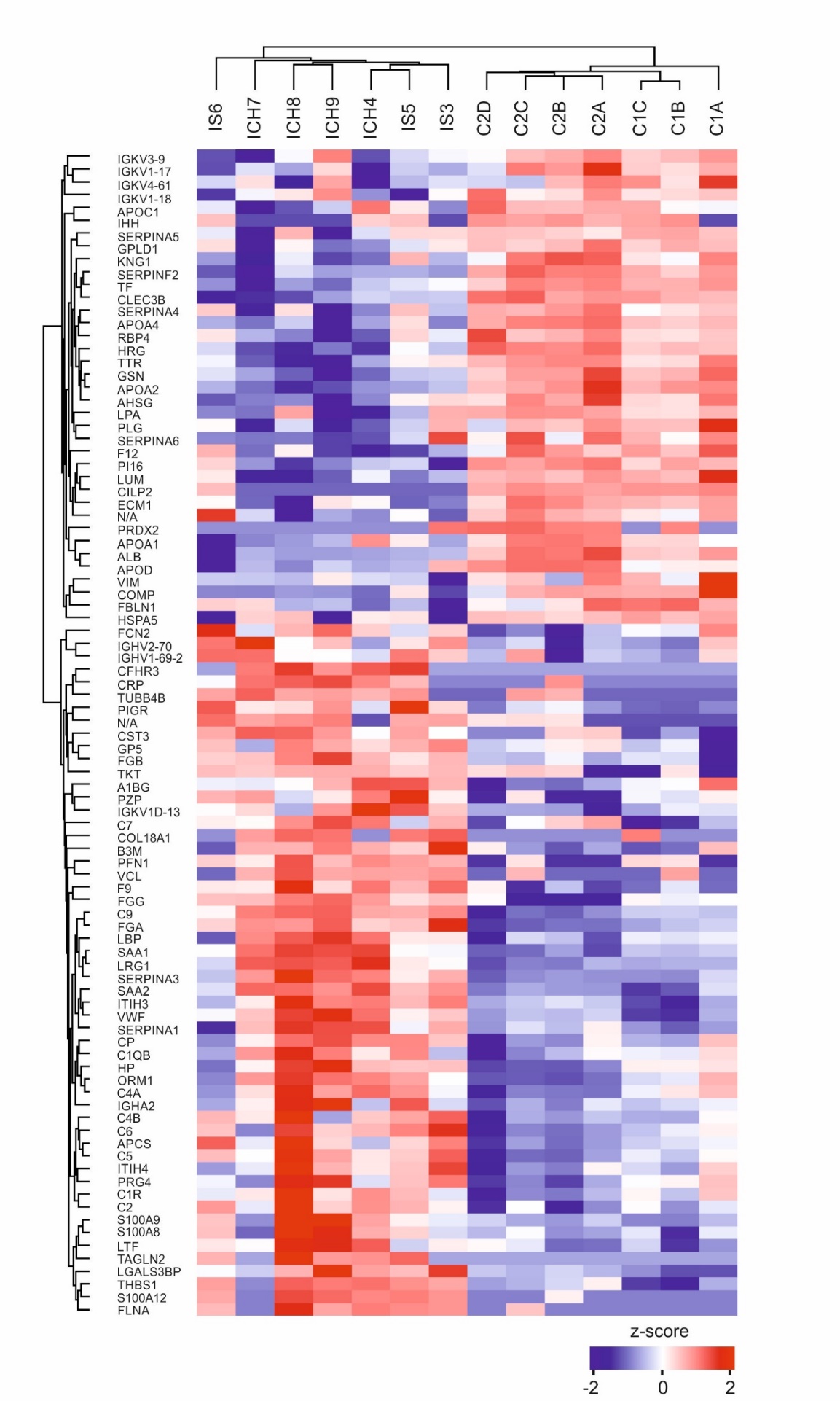


**Supplementary Figure 3.** The heatmap provides an overview of all significantly different expressed proteins (rows) across all patient groups (columns). The results of hierarchical clustering on both protein (rows) and sample (columns) level are indicated on the left and top side of the heatmap, respectively (distance: Euclidean). Deep blue and deep red blocks, respectively, represent very low and very high expression of proteins relative to the average, whereas white blocks indicate no difference in expression.
